# Monoseeding Increases Peanut (*Arachis hypogaea* L.) Yield by Regulating Shade-Avoidance Responses and Population Density

**DOI:** 10.3390/plants10112405

**Published:** 2021-11-08

**Authors:** Tingting Chen, Jialei Zhang, Xinyue Wang, Ruier Zeng, Yong Chen, Hui Zhang, Shubo Wan, Lei Zhang

**Affiliations:** 1 College of Agriculture, South China Agricultural University, Guangzhou 510642, China; chentingting@scau.edu.cn (T.C.); wangxinyuescau@163.com (X.W.); ruierzeng@126.com (R.Z.); chenyong@scau.edu.cn (Y.C.); huizhang@scau.edu.cn (H.Z.); 2 Institute of Crop Germplasm Resources, Shandong Academy of Agricultural Science, Jinan 250100, China; zhangjialei19@163.com

**Keywords:** *Arachis hypogaea* L., monoseeding, shade-avoidance responses, phytochromes

## Abstract

We aimed to elucidate the possible yield-increasing mechanisms through regulation of shade-avoidance responses at both physiological and molecular levels under monoseeding. Our results revealed that monoseeding decreased the main stem height but increased the main stem diameter and the number of branches and nodes compared to the traditional double- and triple-seeding patterns. The chlorophyll contents were higher under monoseeding than that under double- and triple-seeding. Further analysis showed that this, in turn, increased the net photosynthetic rate and reallocated higher levels of assimilates to organs. Monoseeding induced the expression patterns of Phytochrome B (Phy B) gene but decreased the expression levels of Phytochrome A (Phy A) gene. Furthermore, the *bHLH* transcription factors (*PIF 1* and *PIF 4*) that interact with the phytochromes were also decreased under monoseeding. The changes in the expression levels of these genes may regulate the shade-avoidance responses under monoseeding. In addition, monoseeding increased pod yield at the same population density through increasing the number of pods per plant and 100-pod weight than double- and triple-seeding patterns. Thus, we inferred that monoseeding is involved in the regulation of shade-avoidance responsive genes and reallocating assimilates at the same population density, which in turn increased the pod yield.

## 1. Introduction

The global population is expected to reach 8 billion by 2025, which will double future food demand [1]. To meet this demand, crop yield must be increased without increasing the cultivated area. Previously, many studies have been carried out on upgrading crop yield and quality by researchers [2], such as increasing plant population density and nitrogen fertilizer use. However, a suitable population structure requires not only sufficient individuals per unit area but also the rational distribution and uniform development of individuals in the field for maximum utilization of natural resources [3]. Crops grown with high population density that exceed a certain threshold will encounter competition from neighboring vegetation, which restrains plant growth and yield due to limited light, water, and nutrients [4,5].

The alteration of photomorphogenic plant responses to plant population density could be used to increase the yield [6]. The critical variable regulating plant growth and yield at different plant population densities is light [7]. Light is an absolutely necessary resource for crops to carry out photosynthesis. Shade avoidance and shade tolerance are the two contrasting strategies adopted by plants in response to competition for light. Plants perceive low photosynthetically active radiation (PAR) as an early signal of neighbor competition through phytochrome photoreceptors, which in turn induces the shade-avoidance responses (SAR) [8,9]. Generally, a SAR involves the elongation of internodes, reduction in branches, or decrease in leaf number, chlorophyll a/b ratio, or photosynthetic rate that leads to the reallocation of assimilates to stem elongation instead of root and leaf growth, and therefore causes significant decrease in yield [10,11,12,13,14]. These responses are regulated by phytochromes (Phy A–E) [15], which sense decreases in the red/far-red ratio in dense populations and initiate the SAR [8]. At low red/far-red (R/FR) ratios, the phytochrome gene decreases that in turn induce PHYTOCHROME INTERACTING FACTOR (PIF) accumulation [16], which regulates the expression of genes associated with SAR.

Previous studies have attempted to reveal the mechanisms underlying SAR [17,18]. However, the major challenge is to extend our knowledge of this mechanism in plants to develop novel strategies to improve crop yield at high population density. In view of the findings of our investigation and previous research, many useful measures could be implemented to minimize the effect of SAR on crops [19]. The pepper plants were taller and there were fewer branches in double to triple the normal plant population density than normal plant population [20]. Therefore, increased within-row spacing may be a useful measure of plant stand establishment through initiating the effect of SAR, which enables farmers to increase the harvest index or produce high yield with normal population densities.

The peanut (*Arachis hypogaea* L.) is a leguminous crop and an important source of oil and protein for humans, which is cultivated worldwide in tropical and subtropical regions. In China, peanut is grown on more than 5.0 × 10^6^ ha to ensure the supply of edible oil [21]. Traditional planting patterns mainly involved double- and multi-seed sowing, which lead to plant competition, lodging, and low yield. However, to decrease the competition among plants and increase peanut yield, the Shandong Academy of Agricultural Sciences developed a high-yield cultivation technique for monoseeding precision sowing, which was ranked as the main technology by the Ministry of Agriculture and Rural Affairs for five consecutive years from 2015–2019 and promulgated as the national agricultural industry standard [21]. Many scientists have conducted studies to reveal the yield-increasing mechanisms of monoseeding precision sowing that are involved in ontogenetic development and population structure [21,22,23]. However, we assumed that the monoseeding pattern increased the peanut yield through the regulation of SAR. Therefore, the purpose of this study was to decipher the physiological and molecular yield-increasing mechanism of monoseeding.

## 2. Results

### 2.1. Plant Growth and Development

As shown in Table 1, the number of nodes shown significantly different between 2018 and 2019, while the main stem height, main stem diameter and number of branches were insignificant. The main stem diameter, number of branches, and number of nodes were significantly (*p* < 0.05) different among different growing periods and different seeding patterns (Table 2 and Table 3). However, only the growth stage had a significant effect on the main stem height (Table 2), whereas the seeding pattern showed no significant effect on it (Table 3). The monoseeding treatment resulted in the thickest main stem diameter and the highest number of branches and nodes compared with those in the triple-seeding treatments (Table 3). Non-significant differences were observed in the main stem diameter, number of branched and the number of nodes between the double- and triple-seeding treatments. The effect of the growth stage, treatment, and interaction between year and growth stage, and growth stage and treatment, was significant for the main stem height, main stem diameter, number of branches, and number of nodes (*p* < 0.01), whereas only the year had a significant effect on the main stem height, main stem diameter, and number of nodes (*p* < 0.01). However, the interaction of year × growth stage × treatment had no significant effect on the main stem height, main stem diameter and number of branches but did affect the number of nodes (Table 4).

### 2.2. Chlorophyll Content and Net Photosynthetic Rate

In 2018, the leaf SPAD value was significantly higher than that in 2019 (Table 5). The leaf SPAD value in the maturity stage was significantly higher than that in the flowering and pegging stage (Table 6). The SPAD value and net photosynthetic rate (Pn) in the monoseeding treatment shown the highest value when compared with those in the double- and triple-seeding treatments, respectively (Table 7).

### 2.3. Dry Matter Accumulation

The dry matter accumulation of different organs was significantly different among the three seeding treatments during different growing stages in both years (Table 8, *p* < 0.01). The dry matter accumulation of different organs in the monoseeding treatment was higher than that in the double- and triple-seeding treatments in the same growth period in both years. The effects of growth stage, treatment, and the interactions of year × growth stage and growth stage × treatment on root dry weight, leaf dry weight, and stem and petiole dry weight were significant (*p* < 0.01), whereas only the year has significant effect on root dry weight and stem and petiole dry weight (*p* < 0.01). However, the effect of the interactions of year × treatment and year × growth stage × treatment was not significant for root dry weight, leaf dry weight, and stem and petiole dry weight. There were significant differences in pod weight among the three seeding treatments in both years (*p* < 0.01), but year and year × treatment displayed non-significant impact. Furthermore, the pod weight in the monoseeding treatment increased by 34.04% and 123.38% (2018) and by 29.27% and 109.20% (2019) compared with that of the double- and triple-seeding treatments, respectively.

### 2.4. Expression of Shade-Avoidance Response Genes

**Figure 1:**
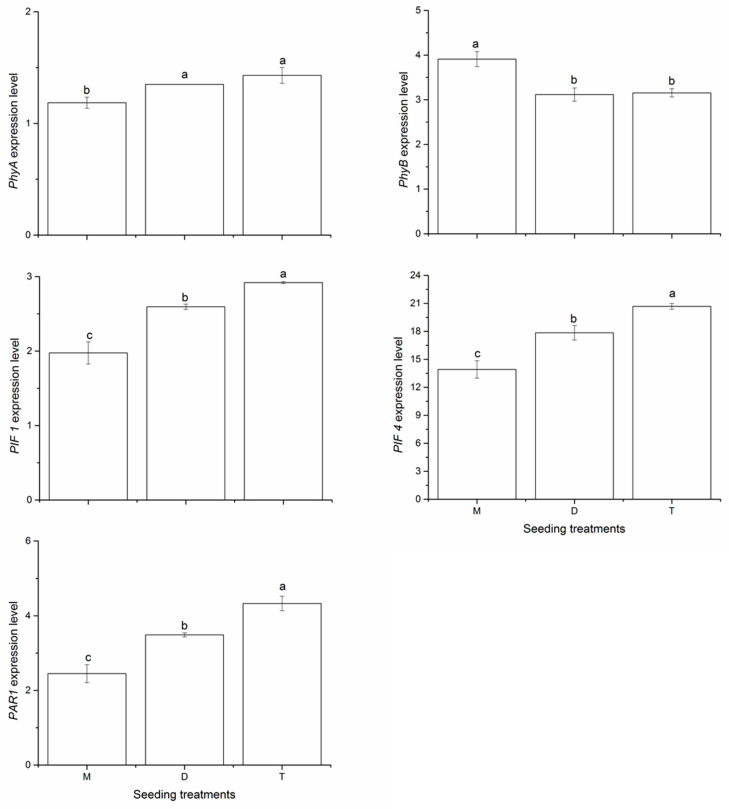
Effects of seeding pattern on the expression of Phy A, Phy B, *PIF 1*, *PIF* 4 and *PAR 1* genes at the flowering and pegging stage in 2018. Mean values marked followed by different letters differ significantly at *p* < 0.05. M, monoseeding, D, double seeding, T, triple seeding.

Five genes that play a major role in crop SAR (Phy A, Phy B, *PIF 1*, *PIF 4*, and *PAR1*) were selected to verify peanut response to shade avoidance. As shown in Figure 1, at the flowering and pegging stage, monoseeding increased the expression of Phy B by 25.52% and 23.93% in 2018, compared with the double- and triple-seeding treatments, respectively. However, the monoseeding treatment significantly decreased the expression of Phy A by 13.92% and 20.67%, compared with the double- and triple-seeding treatments, respectively. We also observed similar effects of seeding pattern on the expression of *PIF 1*, *PIF 4*, and *PAR1* genes. Monoseeding decreased the expression of *PIF 1* by 23.89% and 32.36%, of *PIF 4* by 22.04% and 32.73%, compared with the double- and triple-seeding treatments, respectively. In addition, the expression of *PAR1* in the monoseeding treatment decreased by 29.80% and 43.43%, compared with the double- and triple-seeding treatments, respectively.

### 2.5. Yield and Yield Components

As the seeding number increased, the pod yield significantly decreased (*p* < 0.01) (Table 9). The pod yield in the monoseeding treatment was higher than that in the double- and triple-seeding treatments by 13.68% and 32.04%, respectively. Significant differences were also observed on yield components among seeding treatments. The pod number per plant, 100 pod weight and shelling percentage in the monoseeding treatment shown the significantly highest when compared to those in the double- and triple-seeding treatments.

## 3. Discussion

The present study revealed that monoseeding might be a useful strategy to minimize the SAR of peanut at the same population density as used for the traditional seeding methods and thus increase peanut yield. Monoseeding decreased the main stem height but increased the main stem diameter, number of branches and nodes, SPAD values, and Pn, which is similar to the results in both herbaceous and woody species [24,25,26]. Higher yield was achieved through increasing the number of pods per plant, 100-pod weight, and shelling percentage in the monoseeding treatment. Furthermore, the expression levels of SAR genes were also found to be associated with monoseeding.

Many researchers have found that yield can be increased by minimizing the SAR in crops [12,27]. Here, main stem height decreased but main stem diameter, number of branches, and number of nodes increased compared to the traditional seeding patterns (Table 1), which reduces the competition among plants. Similarly, another study revealed that monoseeding reduces the competition among individuals at the same population density [21]. Moreover, the leaf and root dry biomass were simultaneously reduced in the multiple seeding groups as a result of the reallocation of resources due to the low R/FR ratio [15,28]. We found that the dry matter of different organs in the monoseeding treatment was higher than that in the double- and triple-seeding treatments. This result may be due to the increased reallocation of assimilates to the organs rather than stem elongation compared with that under the traditional seeding patterns.

Leaf chlorophyll content reduction is another phenomenon of SAR [8]. When the R/FR ratio is low, chlorophyll synthesis decreased and the plant accumulates less chlorophyll, which is partly mediated by phytochromes. The response of phytochromes to FR and R radiation plays an important role in adjusting the SAR at high population density [29,30]. Phytochromes are encoded by a small gene family (Phy A, Phy B, and Phy C) in angiosperms, which interact with bHLH transcription factors (PIFs) to control many aspects of photomorphogenesis [31].

Under shaded conditions, the pool of PIFs increases, which regulates the gene expression that promotes the SAR [32]. However, the expression of *PIF 1* and *PIF 4* under monoseeding significantly decreased compared to that in the double- and triple-seeding treatments in our study. This result indicated that monoseeding might reduce the shade for peanut neighbors, enabling plants to absorb more R light and thereby inhibiting the SAR at the same population density as used for the traditional seeding patterns. The decrease in PIFs observed at high PAR was accompanied by an increase in Phy B, which plays a major role in SAR inhibition [9]. We also found that expression of Phy B was increased and *PIF 1* and *PIF 4* expression levels were decreased in the monoseeding treatment, thereby inhibiting the SAR in peanut. These results are in accordance with those of Franklin [33] regarding *Arabidopsis*. Therefore, the regulation of SAR under monoseeding could be due to the decreased expression of *PIF 1* and *PIF 4* and the increased expression of Phy B. However, in the double-seeding treatment with low R/FR, the phytochrome photo-equilibrium shifted to the inactive Pr forms, which no longer interact with *PIF 4* and promote the SAR.

Phy A is the only phytochrome to rapidly decrease at a high R/FR ratio [34]. Previous research indicated that Phy A can reduce the SAR at a low R/FR ratio [35]. In our study, the expression of Phy A significantly decreased in the monoseeding treatment compared to that in the double-seeding treatment, indicating that plants under monoseeding might receive more R radiation from sunlight and convert it into the biological active Pfr form, which interacts with *PIF 4*, triggering additional phosphorylation and alleviating SAR.

PAR was detected initially as an early repressed gene in the photoreceptor signaling pathways and acts as a negative factor of the SAR [36]. At a low R/FR ratio, the expression of *PAR 1* and *PAR 2* increases, which suppresses several auxin-mediated SARs [37]. In contrast, the expression of *PAR1* decreased in the monoseeding treatment compared to that in the double-seeding pattern in our study, suggesting that monoseeding induce the SAR through the low expression level of *PAR1*.

Previous studies have shown that yield remains stable as the plant population reaches the extent of the traditional seeding patterns [38,39]. However, the peanut yield record (10,500 kg ha^−1^), which lasted for 8 years under the traditional seeding pattern, has been broken using the monoseeding pattern at the Shandong Academy of Agricultural Science in 2015 [21]. In our study, monoseeding at the same population density as used for the traditional seeding patterns increased pod number per plant and 100-pod weight, and thus achieved higher pod yield than double-seeding pattern. A probable reason for this that monoseeding reduced the competition among individuals and increased the light received by the plants, which in turn induced the SAR in peanut plants. In our study, monoseeding improved the stand establishment through regulating the SAR and thus produced higher pod yields. Seeds should be dried before shell-peeling and carefully selected for bright color and high plumpness. The above results suggested that monoseeding might be an important change in peanut planting pattern in China and will be widely promoted in the future.

## 4. Materials and Methods

### 4.1. Experimental Design

The field experiments were carried out during 2018 and 2019 at the experimental station of South China Agricultural University in Guangzhou (23°5′ N, 113°23′ E), Guangdong, China. The area has a tropical ocean monsoon climate with an average 21.9 °C annual temperature and 1780 h annual sunlight. The annual average rainfall and evaporation are 1696 and 1591 mm, respectively. A commercial peanut cultivar (*Arachis hypogaea* ‘Huayu 22’) was selected in this study, which is grown at large scale in China. Plants were spaced with 40 cm between rows, 10 cm within rows for monoseeding (planting one seed at a hole, M), and 20 cm within rows for double- (planting two seed at a hole, D) and triple-seeding (planting three seed at a hole, T), which generated three treatments, i.e., plant density of about 25,000 ha^−1^ for M, 25,000 ha^−1^ for D, and 37,500 ha^−1^ for T (Figure S1). A randomized block design with three replications was conducted in our experiments. Each plot consisted of six 10 m rows.

Seeding was conducted on 8 March 2018 and 7 March 2019. Peanut seeds were dropped into the hole at a distance of 10 cm within rows for monoseeding, and 20 cm within rows for double- and triple-seeding. A compound fertilizer, which consists of 81 kg ha^−1^ N, 81 kg ha^−1^ P_2_O_5_, and 81 kg ha^−1^ K_2_O, was used before sowing. Disease, weeds, and pests were controlled according to local agronomic practices.

### 4.2. Data Collection

#### 4.2.1. Plant Traits

At the seedling, flowering and pegging, pod filling, and mature stages, 10 labeled plants were selected from each plot, and the main stem height, stem diameter at 10 cm, number of branches, and number of main stem nodes were recorded.

#### 4.2.2. Dry-Matter Accumulation

We collected six plant samples from each plot at the seedling, flowering and pegging, and mature stages, respectively. The plant samples were separated into leaves, roots, and stems. Each fresh organ was dried at 105 °C for 30 min followed by 80 °C to a constant dry weight.

#### 4.2.3. Chlorophyll Content and Photosynthetic Parameters

The SPAD value and photosynthetic parameters were determined for six selected plants from each plot at the flowering and pegging (24 April 2018, and 25 April 2019), and pod filling stages (15 June 2018, and 15 June 2019). Due to the amount of rainfall prior to the measurement days (23 April (10.0 mm) and 13 June (22.4 mm) in 2018, 22 April (24.4 mm) and 13 June (14.8 mm) in 2019, data from the weather station in our experiment station), the soil in the fields was wet during the measurements. The SPAD value in the leaves (third upper fully expanded leaves of the main stem) was determined using a chlorophyll meter (SPAD-502, Konica Minolta Sensing Inc., Osaka, Japan). The net photosynthetic rate (Pn) of the third upper fully expanded leaves was measured using a LI-6400 portable photosynthesis system (LI-COR, Lincoln, NE, USA) with a 6 cm^2^ leaf-area chamber by using a red-blue LED array (6400-02B) between 9:00 and 11:00 a.m. The measurement conditions inside the leaf chamber were kept constant (light intensity was at 1400 µmol m^−2^ s^−1^, and the internal CO_2_ concentration was at 400 µmol mol^−1^). The leaf temperature (measured by a thermocouple inside the chamber) ranged from 28.40 to 31.60 °C and the vapor pressure deficit, which was calculated based on the above leaf temperature and air temperature, ranged from 1.46 to 2.49 kPa. The data were recorded after the gas exchange parameters stabilized (about 3–5 min).

#### 4.2.4. RNA Extraction and qRT-PCR Analysis

Total RNA was extracted from 250 mg fresh leaves of each plot at the flowering and pegging stage using the TRIzol reagent (Invitrogen, USA ). Then 2μg of RNA was reverse transcribed to cDNA with SuperScriptIII RTS First-Strand cDNA Synthesis Kit (Thermo Fisher, China). Five genes (Phy A, Phy B, *PIF 1*, *PIF4*, and *PAR 1*) related to the SAR were retrieved from *A. hypogaea* database (peanutbase.org, version KYV3) [40] and *UBI 2* was used as a reference gene which reported by Luo et al. [41]. Primer pairs were designed through primer-BLAST (https://www.ncbi.nlm.nih.gov/tools/primer-blast/(accessed on 30 January 2020)) using the following parameters: PCR product size between 100 and 200 bp; melting temperature (Tm) between 57 and 63 °C; (Table S1). Thermal cycling was run on a BIO-RED IQ2 Sequence Detection System at a denaturation step at 94 °C for 3 min, 35 cycles (94 °C for 30 s, 60 °C for 30 s, and 72 °C for 25 s, followed by one step at 72 °C for 10 min. CT values were obtained through analyzing amplification plots with a 0.2 fluorescence signal threshold. All CT values of genes were normalized to the CT value of the UBI2 gene. The PCR efficiency (E) was calculated according to the method of Ramakers et al. [42]. The gene of interest (GOI) was calculated as: GOI = (1 + E)^−ΔCT^, where ΔCT = CT_GOI_ − CT_reference_.

#### 4.2.5. Peanut Yield and Yield Components

At harvest, 1.25 m of four rows was delimited in each plot and the pod yields were determined. Six consistent plants were sampled from each plot to count the number of pods per plant. All pods from the peanut plants were collected and air-dried for 15 days. The 100-pod weight and shelling percentage were measured according to Zhang et al. [43].

### 4.3. Statistical Analysis

Data processing was conducted in SPSS 16.0 (SPSS, Chicago, IL, USA). All data are presented as the mean (± SD) of six replicates. The difference between mean values greater than the least significant difference (LSD) (*p* = 0.05) was considered as significant. A three-way analysis of variance (ANOVA) with a randomized block design was used to assess the effect of treatments. Originpro 9.0 was used for drawing figures.

## 5. Conclusions

Monoseeding at the same population density as traditional seeding patterns reduced the main stem height but increased the main stem diameter, number of branches and nodes, and dry matter accumulation via the rapid upgraded chlorophyll content and net photosynthesis rate. Furthermore, the Phy B expression increased, and concomitantly, the expression of Phy A, *PIF 1*, *PIF4*, and *PAR 1* decreased in the monoseeding treatment in our study. These changes coordinated with plant responses might explain the improved growth of peanut plants in monoseeding through regulating shade avoidance responses. Monoseeding increased the pod yield through upgrading the pod number per plant and 100-pod weight compared with the traditional seeding pattern. The overall results suggested that monoseeding at the same population density as used for traditional seeding methods represents a novel alternative seeding pattern able to increase the pod yield for peanut production by regulating SAR.

## Figures and Tables

**Table 1:** Effect of different years on plant growth parameters of peanut.

Years	Parameters			
	Main Stem Height (cm)	Main Stem Diameter (mm)	Number of Branches	Number of Nodes
2018	26.8 ± 10.9 a	3.1 ± 1.2 a	5.7 ± 2.8 a	6.7 ± 2.6 a
2019	25.3 ± 11.8 a	3.1 ± 1.3 a	5.6 ± 2.7 a	7.9 ± 2.4 b

Mean values within a column followed by different letters are significantly different at *p* < 0.05, on the basis of LSD test.

**Table 2:** Effect of different growth stages on plant growth parameters of peanut.

Years	Parameters			
	Main Stem Height (cm)	Main Stem Diameter (mm)	Number of Branches	Number of Nodes
Seedling stage	14.5 ± 2.4 d	1.9 ± 0.2 c	3.1 ± 1.5 c	4.8 ± 1.4 b
Flowering and pegging stage	19.8 ± 3.9 c	2.4 ± 0.5 c	5.0 ± 1.9 bc	6.1 ± 1.2 b
Pod filling stage	28.0 ± 3.5 b	3.4 ± 0.7 b	6.1 ± 2.0 ab	8.3 ± 1.8 a
Maturity stage	41.6 ± 6.5 a	4.6 ± 0.9 a	8.3 ± 2.5 a	9.9 ± 1.8 a

Mean values within a column followed by different letters are significantly different at *p* < 0.05, on the basis of LSD test.

**Table 3:** Effect of seeding pattern on plant growth parameters of peanut.

Treatment	Main Stem Height (cm)	Main Stem Diameter (mm)	Number of Branches	Number of Nodes
M	22.2 ± 9.3 a	3.8 ± 1.4 a	7.9 ± 2.5 a	8.9 ± 2.5 a
D	25.7 ± 10.9 a	3.1 ± 1.1 ab	5.5 ± 2.1 b	7.1 ± 2.3 ab
T	30.1 ± 13.1 a	2.4 ± 0.8 b	3.6 ± 1.6 b	5.8 ± 1.8 b

M, monoseeding, D, double seeding, T, triple seeding. Mean values within a column followed by different letters are significantly different at *p* < 0.05, on the basis of LSD test.

**Table 4:** Mean square of ANOVA of the effect of year, growth stage, seeding pattern and their interaction on plant growth parameters.

	Parameters			
	Main Stem Height (cm)	Main Stem Diameter (mm)	Number of Branches	Number of Nodes
Year (Y)	49.9 **	0.0 **	0.2 NS	10.9 **
Growth stage (G)	2464.1 **	17.8 **	114.5 **	83.2 **
Treatment (T)	379.1 **	6.9 **	148.8 **	83.0 **
Y×G	53.2 **	0.1 **	1.2 **	3.7 **
Y×T	0.1 NS	0.0 **	4.1 **	2.7 NS
G×T	29.1 **	0.5 **	2.0 **	1.0 **
Y×G×T	0.7 NS	0.0 NS	0.2 NS	2.5 **

** significant at the *p* < 0.01 levels, respectively. NS, non significant.

**Table 5:** Effect of different years on leaf SPAD value of peanut.

Years	SPAD
2018	41.2 ± 2.5 a
2019	37.2 ± 4.0 b

Mean values within a column followed by different letters are significantly different at *p* < 0.05, on the basis of LSD test.

**Table 6:** Effect of different growth stages on leaf SPAD value of peanut.

Growth Stages	SPAD
Flowering and pegging stage	37.4 ± 4.5 b
Maturity stage	40.8 ± 2.2 a

Mean values within a column followed by different letters are significantly different at *p* < 0.05, on the basis of LSD test.

**Table 7:** Effect of seeding pattern on leaf chlorophyll content and net photosynthesis rate of peanut.

Treatment	SPAD	Net Photosynthetic Rate(Pn) (μmol CO_2_ m^−2^ s^−1^)
M	41.9 ± 2.9 a	21.5 ± 1.2 a
D	38.6 ± 3.5 b	19.4 ± 0.7 b
T	36.3 ± 3.4 b	16.8 ± 0.7 c

M, monoseeding, D, double seeding, T, triple seeding. Mean values within a column followed by different letters are significantly different at *p* < 0.05, on the basis of LSD test.

**Table 8:** Effect of seeding pattern on dry matter accumulation of peanut at different growth stages in 2018 and 2019.

Year	Growth Stage	Treatment	Root Dry Weight (g/Plant)	Stem and Petiole Dry Weight (g/Plant)	Leaf Dry Weight (g/Plant)	Pod Dry Weight (g/Plant)
2018	Seedling stage	M	0.9 ± 0.0 a	4.6 ± 0.2 a	6.8 ± 0.4 a	-
		D	0.6 ± 0.0 b	3.9 ± 0.0 b	5.6 ± 0.4 b	-
		T	0.4 ± 0.0 c	3.1 ± 0.3 c	4.7 ± 0.1 c	-
	Flowering and pegging stage	M	1.0 ± 0.1 a	7.4 ± 0.1 a	11.6 ± 0.3 a	-
		D	0.8 ± 0.0 b	5.7 ± 0.0 b	9.8 ± 0.2 b	-
		T	0.6 ± 0.0 c	4.6 ± 0.4 c	7.7 ± 0.1 c	-
	Maturity stage	M	3.3 ± 0.2 a	16.3 ± 0.7 a	14.1 ± 0.5 a	44.1 ± 0.6 a
		D	2.4 ± 0.2 b	12.2 ± 0.3 b	11.9 ± 0.2 b	32.9 ± 3.6 b
		T	1.8 ± 0.1 c	10.1 ± 0.7 c	9.1 ± 0.3 c	19.8 ± 2.5 c
2019	Seedling stage	M	0.5 ± 0.0 a	2.9 ± 0.1 a	4.1 ± 0.2 a	-
		D	0.3 ± 0.0 b	2.2 ± 0.2 b	3.1 ± 0.1 b	-
		T	0.3 ± 0.0 c	1.7 ± 0.0 c	2.0 ± 0.3 c	-
	Flowering and pegging stage	M	1.3 ± 0.1 a	7.8 ± 0.0 a	9.3 ± 1.1 a	-
		D	1.0 ± 0.0 b	5.2 ± 0.1 b	6.4 ± 0.2 b	-
		T	0.7 ± 0.1 c	3.9 ± 0.0 c	4.9 ± 0.1 c	-
	Maturity stage	M	2.9 ± 0.0 a	18.3 ± 1.3 a	13.6 ± 0.2 a	43.6 ± 3.8 a
		D	2.1 ± 0.1 b	14.8 ± 0.0 b	12.0 ± 0.3 b	33.8 ± 0.8 b
		T	1.6 ± 0.0 c	11.9 ± 0.1 c	8.8 ± 0.8 c	20.9 ± 1.3 c
			Mean Square			
Year (Y)			0.1 **	0.1 NS	32.2 **	0.1 NS
Growth stage (G)			11.4 **	384.2 **	154.7 **	-
Treatments (T)			1.8 **	41.4 **	41.6 **	562.7 **
Y × G			0.2 **	10.4 **	6.2 **	-
Y × T			0.0 NS	0.1 NS	0.0 NS	0.7 NS
G × T			0.3 **	6.4 **	2.2 **	-
Y × G × T			0.0 NS	0.2 NS	0.2 NS	-

M, monoseeding, D, double seeding, T, triple seeding. Mean values within a column followed by different letters are significantly different at *p* < 0.05, on the basis of LSD test. Mean square (MS) for all these main effects and interactions are shown. -, means that the data of pod cannot be collected during the nutrition growth stage, ** significant at the *p* < 0.01 levels. NS, non-significant.

**Table 9:** Effect of seeding pattern on yield and yield component of peanut.

Treatment	Pods Number per Plant	100 Pod Weight (kg)	Shelling Percentage (%)	Pod Yield (kg/hm^2^)
M	23.61 ± 0.49 a	0.21 ± 0.01 a	76 ± 0.76 a	11,683.75 ± 145.58 a
D	19.15 ± 0.70 b	0.19 ± 0.01 b	73 ± 0.59 b	10,277.00 ± 290.87 b
T	14.52 ± 3.48 c	0.16 ± 0.01 c	70 ± 0.74 c	8848.75 ± 238.05 c

M, monoseeding, D, double seeding, T, triple seeding. Mean values within a column followed by different letters are significantly different at *p* < 0.05, on the basis of LSD test.

## Data Availability

All data have been presented in the manuscript and Supplementary Materials, so the study did not report other data.

## Supplementary Materials

The following are available online at https://www.mdpi.com/article/10.3390/plants10112405/s1, Figure S1: Cultivation schematic model of peanut in the field, Table S1: Primers used for qRT-PCR analysis.
