# Supplementary material for: Monoseeding Increases Peanut (Arachis hypogaea L.) Yield by Regulating Shade-Avoidance Responses and Population Density
Source: Plants (Basel). 2021 Nov 8;10(11):2405. doi: 10.3390/plants10112405 (PMC8625293; doi:10.3390/plants10112405)
Supplement: Supplementary file 1 [file plants-10-02405-s001.zip › plants-1328548-supplementary.pdf]

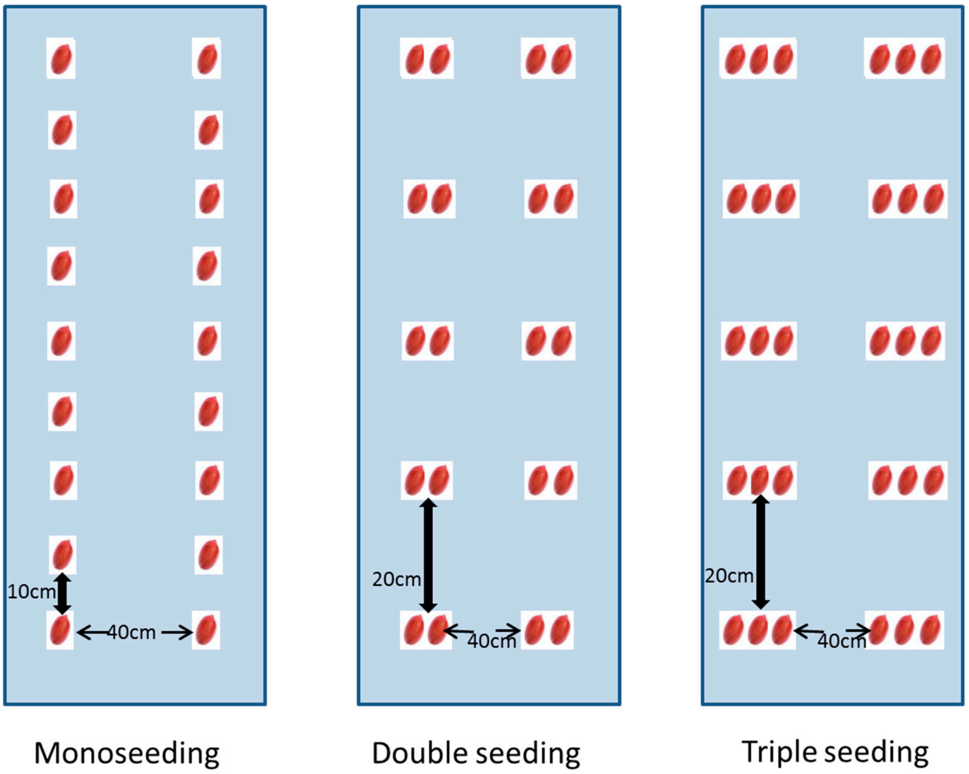

Figure S1 Cultivation schematic model of peanut in the filed

Table S1 Primers used for qRT-PCR analysis

Gene name/Gene ID	Forward primer(5'-3')	Reverse primer (5'-3')
UBI2/HO115753.1	AAGCCGAAGAAGATCAAGCAC	GGTTAGCCATGAAGGTTCCAG
Phy A/XM_025819628.2	GCTGATGAATGGAGATGTGCG	CGTGACAACAACCGACCTTT
Phy B/ NC_037621.1	CGACGCGTCAATAAGCAAGG	ACGGAGTGGGAGTAGTCGAA
PIF1/XM_029290071.1	ATCAAACCGGCAGAGTGGAG	GGCACTTGTGGATGGTCTGA
PIF4/XM_025812414.2	AATCCTATGCCCGCTCCAAG	AACTTTTGTTGTGCGGGTGC
PAR1/XM_025846249.1	ATTGCTACCAAGCATGCCCT	AGGTGCCACAATACCAGAGC
